# Squaring the Circle: AIDS, Poverty, and Human Development

**DOI:** 10.1371/journal.pmed.0040314

**Published:** 2007-10-23

**Authors:** Peter Piot, Robert Greener, Sarah Russell

## Abstract

The authors discuss the "downstream" effects of AIDS on poverty, and the "upstream" effects of poverty upon acquiring HIV.

It is often asserted that AIDS is at the core of a “vicious circle”, whereby the impacts of AIDS increase poverty and social deprivation, while poverty and social deprivation increase vulnerability to HIV infection. In examining this view, it is important to distinguish between what might be called the “downstream” effects of AIDS on poverty, and the “upstream” effects of poverty upon the risk of acquiring HIV.

## The “Upstream Effects”: Poverty and Vulnerability to HIV

Generally speaking, there is a strong association between poverty and ill health—wealthier countries and wealthier individuals enjoy better health as measured by a variety of indicators such as life expectancy or incidence of waterborne diseases. Many researchers have had the same expectation about AIDS, which has often been described as a “disease of poverty” [1].

There is one fundamental difference, however, between AIDS and other health problems generally linked with poverty. Unlike diseases such as tuberculosis and malaria, HIV is mostly transmitted through sex. This brings into play the economic perspective around reward and dependency, which influences the extent to which individuals are able to make and exercise choices about sexual behaviour. Recent evidence clearly indicates that AIDS is a disease of inequality, often associated with economic transition, rather than a disease of poverty in itself.

Undeniably, more people live with HIV in poor countries than in rich ones. More than 60% of people living with HIV inhabit the world's poorest region: sub-Saharan Africa. Nevertheless, studies during the early stage of the epidemic suggested that HIV incidence initially occurred not amongst the poorest, but among better-off members of society in this region. A decade later, infections still appear more concentrated among the urban employed and more mobile members of society, and consequently the more wealthy groups, as can be seen in Table 1 [2].

**Table 1:**
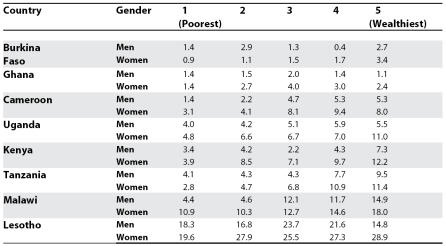
HIV Prevalence by Wealth Quintile in Eight African Countries (Percentage of Adult Population)

It is important to note that HIV prevalence is very high in all of the wealth quintiles—including quintile 1, the poorest (the wealthiest is quintile 5). While there is abundant research that documents the mechanisms that lead from extreme poverty to HIV vulnerability [3], the data now indicate that the less well-studied risk factors among the better-off in fact dominate the aggregate picture. The fact that most people living with HIV in the region today are poor simply reflects the fact that the epidemic has now spread throughout the generalized population in a region that has a high proportion of poor people.

Whether the patterns observed in this part of Africa will also emerge elsewhere remains to be seen. In Asia, for example, epidemics have so far been mostly concentrated in sex workers (and their clients) and drug users, who are often very poor. Infection patterns are now influenced by rapid economic development, which has increased the movement of both men and women in search of opportunities within and across borders. And here, as in Africa, mobility correlates to higher rates of HIV infection.

Many researchers now point not to poverty itself but to economic and gender inequalities and weakened “social cohesion” [4] as factors influencing sexual behaviour and hence the potential for HIV transmission. Some, notably Amartya Sen [5], have looked at poverty as an outcome of poor governance. It has been suggested that, by extension, regimes that do not focus on the well-being of populations impoverish their citizens, deny their enjoyment of basic human rights, and erode public health—exacerbating both the upstream and downstream effects of AIDS. This assertion clearly warrants further research.

One indication of the connection between poor governance and HIV is the clear pattern of association between income inequality as measured by the Gini coefficient and HIV prevalence across countries in sub-Saharan Africa—countries with greater inequality have higher HIV prevalence (Figure 1—using HIV prevalence and economic data from 2006) [6]. However, some of the countries with the highest inequality and HIV prevalence, such as Botswana, South Africa, and Namibia, have low levels of domestic corruption as measured by the regional corruption perceptions index published by Transparency International (http://www.transparency.org/policy_research/surveys_indices/cpi), and are regarded as having reasonably good governance. Meanwhile, some countries that have high levels of corruption according to this index, such as Kenya and Côte D'Ivoire, have lower—but still very significant—HIV prevalence, suggesting that inequality is a stronger predictor of HIV prevalence than poor governance. One possible consequence of this apparent association between inequality and HIV is that economic growth that is not pro-poor and that leads to greater income inequality may even fuel the HIV epidemic.

**Figure 1:**
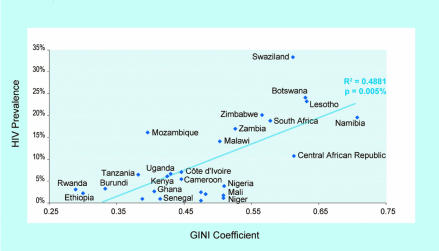
HIV Prevalence and Income Inequality in Africa The Gini coefficient has a value between 0 and 1, representing the extremes of income distribution. A zero value corresponds to the situation where everyone in the population has exactly the same income, whereas a value of 1 would correspond to extreme concentration of income in one person. A high value indicates a more unequal income distribution. Note that Figure 1 uses data from only one year and therefore cannot show any dynamic relationship between changes in income inequality (which are slow), and changes in HIV prevalence.

This result also appears to be applicable to gender income inequality—at household level and more generally. Where women's economic and social safety is largely dependent on their partners' occupations and status, they have little choice in determining their own sexual safety [7]. A study in Kenya found that higher gender inequality between young women and adult men is significantly and strongly correlated to positive HIV status (K. Beegle, unpublished data).

Gender inequality is one of many injustices fuelling the epidemic. The spread of HIV is disproportionately high among many groups that experience discrimination and suffer from a lack of human rights protection. This includes groups that have been marginalized socially, culturally, and often economically, such as injection drug users, sex workers, migrants, and men who have sex with men. Women's susceptibility to HIV is further enhanced in members of marginalized or migrant populations: research in Viet Nam, for example, revealed that women migrant workers were twice as likely as other women to become HIV positive [8].

## The “Downstream Effects”: The Impact of AIDS on Poverty and Development

The economic impacts of AIDS are proportionately greater for poor households, and AIDS can be expected to increase both poverty and income inequality. At the same time, AIDS has a significant impact on the aggregate economy of high-prevalence countries.

AIDS kills people in the prime of their working and parenting lives, with a devastating effect on the lives and livelihoods of affected households. Incomes shrink when employed household members become sick or die, and resources are further depleted by medical and funeral-related costs. The impact on poor households is clearly disproportionate, with many struggling to meet demands for treatment and care. (For a comprehensive survey of literature on HIV impacts, see [9].) For example, in India, the financial burden on households living with HIV was 82% of income in the poorest quintile and just over 20% among the richest quintile [10]. The very poor struggle to afford even heavily subsidized antiretroviral treatment. This struggle is particularly acute for women. Although more than half of all adults living with HIV in Zambia are women, when the monthly cost for HIV treatment was reduced from US$64 to US$8 as a result of funding from the Global Fund to Fight AIDS, Tuberculosis and Malaria, many more men than women showed up for treatment. In one rural town, of the 40 adults taking antiretroviral drugs, only three were women. Moreover, even if drugs are free, poor families may have insufficient resources to meet basic nutrition needs or the costs of travel to health clinics for care [11].

We can thus expect AIDS to impede efforts to reduce poverty. In high-prevalence countries such as Botswana, estimates have projected that the number of households living in poverty will increase at a rate that is 0.5% higher per year than if there were no AIDS [12]. A smaller effect can be expected in lower-prevalence countries, but the number of households living in poverty is likely to increase by at least 0.1%–0.5% per year.

The epidemic is dramatically increasing the numbers of orphans, particularly in high-prevalence countries. More than 15 million children worldwide have been orphaned by AIDS—over 12 million of them in sub-Saharan Africa, overwhelming the capacity of social networks and traditional patterns of intergenerational dependency, and creating an uneducated, unsocialized, and often uncared-for generation [4].

Companies in high-prevalence countries can also expect AIDS to decrease productivity and increase costs for recruitment and training, with the magnitude of these effects depending on the employment conditions and benefits offered by the company, the HIV prevalence in the workforce, and the skill levels of the employees most affected. A study in Africa has found widely varying HIV-related costs from 0.5%–10% of the total labour costs—which in some sectors would constitute a very significant proportion of company profit (see the examples in Table 2 [J. Simon et al., unpublished data])

**Table 2:**
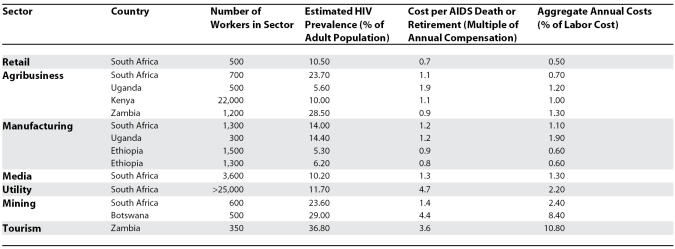
HIV-Related Labor Costs

Government and the public sector are similarly affected by increases in employment costs, but also experience the results of a falling revenue base (to the extent that economic growth is affected), and increased demands for health and other public services related to the epidemic.

At a national level, the macroeconomic impact is expected to be relatively small when compared to that of other factors related to the global economic environment and the quality of economic management in affected countries. Most estimates making use of standard economic models in high-prevalence countries indicate a reduction of about 0.5%–1.5% in the gross domestic product (GDP) growth rate over a 10 to 20-year time frame compared to the situation without AIDS (for a useful summary, see [13]). The impact of tuberculosis—many cases of which are linked to HIV—has been estimated to be $1.4–$2.8 billion per annum, slightly less than half of the AIDS estimates [14], while the impact of malaria has been estimated to be in the same range as AIDS—as much as 1.3% of GDP growth in highly affected countries [15]. This level of impact is significant, therefore, but not catastrophic. Impact in lower-prevalence countries can be expected to be correspondingly lower.

Some researchers have drawn attention to the longer term potential for progressive weakening of human capital, and the lost transmission of knowledge and skills between generations. This is of particular concern given the increasing recognition that AIDS is a long-term phenomenon for which long-term strategies are required.

## Responding to the Interactions between AIDS and Poverty

Understanding both upstream and downstream interactions between AIDS and poverty is critical to understanding local and global epidemiological trends and patterns. Such an understanding is, in turn, vital to the development and implementation of effective strategies to prevent and treat HIV.

Growing awareness of the economic aspects of the epidemic has helped catalyze greater political action on AIDS in recent years. For example, AIDS is the only health issue ever to become the subject of a United Nations Security Council debate or a Special Session of the UN General Assembly. It has been featured regularly at G8 meetings and regional summits in Africa, the Caribbean, and elsewhere. In more than 40 countries, National AIDS Programmes are led by the President, Vice-President, or Prime Minister.

Political action has fuelled financial investment. Global expenditure on AIDS in low- and middle-income countries increased from $250 million in 1996 to an expected $10 billion in 2007.

We are now beginning to see a return on this investment. Fewer people are becoming infected with HIV in almost all East African countries, in the Caribbean, in Cambodia, and in southern India. Almost 2.5 million people are now on antiretroviral therapy in developing countries—up from 100,000 five years ago. To build on this progress and ensure that it is sustainable, however, six elements are key.

**First, AIDS money has the most impact when strategies are based on the concept of “know and act on your epidemic”.** UNAIDS' *Practical Guidelines for Intensifying HIV Prevention* [16] provide practical guidance to tailor national HIV prevention responses so that they respond to the epidemic dynamics and social context of the country and reach populations who remain most vulnerable to HIV infection. For example, economic growth and the growth of trade between neighbouring countries can be expected to lead to increased mobility, particularly of transport workers, which is known to increase vulnerability to HIV in the absence of tailored prevention efforts. In parts of Asia, mobile men who have unprotected sex with sex workers are a key factor in spreading HIV [17].

**Second, a growing number of small-scale activities indicate the value of combining HIV programmes with poverty reduction initiatives.** In Malawi, non-governmental organizations integrate HIV prevention into village banking programmes for women, and combine AIDS education with the provision of microfinance to groups of women through community banking programmes [18]. In Northern Thailand, the Population and Community Development Association's Positive Partnership Project offers loans to partnerships consisting of a person living with HIV and a “buddy” (often a friend or family member who is not living with HIV) to set up small business ventures [19]. The challenge now, however, is to make the shift from small-scale projects to large-scale programmes.

**Third, the provision of HIV treatment can help prevent poverty—and indirectly contribute to HIV prevention as well—by helping to break down stigma.** A study in Kenya observed a rapid improvement in labour productivity among tea pickers in 12 months after starting antiretroviral treatment [20]. Such findings highlight the economic importance of ensuring that the poor have access to HIV treatment and prevention services. This access requires action in four key areas: increased investment in antiretroviral treatment—by both national and international funders; a reduction in the cost of antiretroviral drugs; improved HIV service delivery systems; and better services for the poor.

**Fourth, development plans (whether they concern the development of productive sectors or the provision of social safety nets) must “pass the AIDS test”.** Development initiatives must contribute to AIDS prevention and treatment in the communities they work in. The World Bank–supported Chad/Cameroon Pipeline Project, for example, supports HIV workplace interventions along the pipeline route—both for workers and for affected communities [21].Social protection programmes must also include specific measures to address the economic and social needs of households that are directly affected by AIDS.

**Fifth, both poverty reduction programmes and AIDS strategies must reduce vulnerability to HIV—particularly for women and young people.** Doing so involves protecting human rights and tackling issues around social marginalization and stigma. The link between poverty, gender inequality, and HIV has prompted many institutions to talk about integrating gender and AIDS into development and poverty reduction strategies, but far more action is required [22]. One obvious step is to ensure that domestic legislation is consistent with international human rights norms, and that it is effective in protecting women's rights within marriage, securing their right to own and inherit property, ensuring equality in the workplace, and strengthening laws against domestic violence.

**Sixth, addressing AIDS in the world's poorest countries and communities depends on increased and sustained international support, driven by high-level political will.** Although it is ultimately the responsibility of all states to provide HIV prevention and treatment for all citizens, and to mitigate the impact of AIDS on the poor, many countries require international support to live up to these responsibilities, and are likely to do so for some time to come. Failure by donors to prioritise the provision of such support is likely to undermine the effectiveness of current and future efforts, and could also lead to the unravelling of progress already made.

## Conclusion

AIDS, it has been said, is exceptional [23] in terms of its threat to humanity, and its complexity. The relationship between AIDS and poverty is just one example of that complexity, having more to do with inequality than poverty per se.

Another example of complexity is that the relationship between socioeconomic status and HIV varies considerably from country to country, reflecting differences in culture and traditions. Effective actions to tackle AIDS must directly address these specific factors—the inequalities—that drive HIV transmission in different contexts, and must overcome the obstacles to accessing treatment in different groups. [6].

Complex problems famously require complex solutions. In this case, it is crucial to place AIDS squarely at the centre of all socio-economic development, and provide long-term, high-level domestic and international investment in HIV prevention and treatment in the world's poorest countries.

## Abbreviations

GDP: gross domestic product
